# *ThMYC4E*, candidate *Blue aleurone 1* gene controlling the associated trait in *Triticum aestivum*

**DOI:** 10.1371/journal.pone.0181116

**Published:** 2017-07-13

**Authors:** Na Li, Shiming Li, Kunpu Zhang, Wenjie Chen, Bo Zhang, Daowen Wang, Dengcai Liu, Baolong Liu, Huaigang Zhang

**Affiliations:** 1 Qinghai Province Key Laboratory of Crop Molecular Breeding, Xining, China; 2 University of Chinese Academy of Sciences, Beijing, China; 3 Key Laboratory of Adaptation and Evolution of Plateau Biota (AEPB), Northwest Institute of Plateau Biology, Chinese Academy of Sciences, Qinghai Xining, China; 4 State Key Laboratory of Plant Cell and Chromosome Engineering, Institute of Genetics and Development Biology, Chinese Academy of Sciences, Beijing, China; 5 Triticeae Research Institute, Sichuan Agricultural University, Chengdu, Sichuan, China; New South Wales Department of Primary Industries, AUSTRALIA

## Abstract

Blue aleurone is a useful and interesting trait in common wheat that was derived from related species. Here, transcriptomes of blue and white aleurone were compared for isolating *Blue aleurone 1* (*Ba1*) transferred from *Thinopyrum ponticum*. In the genes involved in anthocyanin biosynthesis, only a basic helix-loop-helix (bHLH) transcription factor, *ThMYC4E*, had a higher transcript level in blue aleurone phenotype, and was homologous to the genes on chromosome 4 of *Triticum aestivum*. *ThMYC4E* carried the characteristic domains (bHLH-MYC_N, HLH and ACT-like) of a bHLH transcription factor, and clustered with genes regulating anthocyanin biosynthesis upon phylogenetic analysis. The over-expression of *ThMYC4E* regulated anthocyanin biosynthesis with the coexpression of the MYB transcription factor *ZmC1* from maize. *ThMYC4E* existed in the genomes of the addition, substitution and near isogenic lines with the blue aleurone trait derived from *Th*. *ponticum*, and could not be detected in any germplasm of *T*. *urartu*, *T*. *monococcum*, *T*. *turgidum*, *Aegilops tauschii* or *T*. *aestivum*, with white aleurone. These results suggested that *ThMYC4E* was candidate *Ba1* gene controlling the blue aleurone trait in *T*. *aestivum* genotypes carrying *Th*. *ponticum* introgression. The *ThMYC4E* isolation aids in better understanding the genetic mechanisms of the blue aleurone trait and in its more effective use during wheat breeding.

## Introduction

Blue grain wheat cultivars have a blue aleurone layer that contains a group of anthocyanin pigments that differ from those in purple, red or white wheat grains [1–3]. Because it is easily observed, the blue aleurone trait has been used as a genetic marker for measuring the outcrossing frequencies among wheat cultivars, detecting the distance of pollen transmission, identifying true hybrids and monitoring chromosomal changes caused by wheat chromosomal engineering [4–8]. A 4E-ms system has been established for producing hybrid wheat in China by tracking the 4E chromosome, which carries a fertility restorer gene, using the blue aleurone trait [9]. Moreover, anthocyanin pigments carry anti-inflammatory, anti-mutagenic, anti-carcinogenic and antibacterial functions, and combat hepatotoxicity and the induction of apoptosis in healthy humans [10, 11]. Thus, blue grain wheat genotype was a potential dietary source of bioactive materials to prevent diseases and promote health in the functional food industry.

No hexaploid wheat with blue grains was described prior to the introgression of genes from *Agropyron* species and diploid wheat [4, 12]. The blue-grain genes from *Th*. *ponticum* (syn. *Agropyron elongatum*) and *T*. *monococcum* were named as the *Blue aleurone 1* (*Ba1*) and *Blue aleurone 2* (*Ba2*), respectively [8, 12]. *Ba1* was physically mapped to the region of FL0.71–0.80 on the long arm of chromosome 4E using a set of translocations that showed different seed colors [8]. The chromosome 4B or 4D could be substituted by chromosome 4E in the disomic substitution lines [7, 8], which suggested 4B, 4D, and 4E were the homologous chromosome. A segregation ratio of 3 blue:1 white was observed in the F_2_ population derived from the cross of blue wheat and white wheat [8]. *Ba1* is incompletely dominant to the non-blue aleurone allele. Grains were dark blue when there were three doses of the 4E chromosome in the endosperm cells, blue when there were two doses, pale blue with one dose, and white in the absence of the 4E chromosome [13, 14]. Genes homologous to *Ba1* existed in the chromosome 4A of *T*. *monococcum* (2n = 2x = 14, genome AA), and 4J of *Th*. *bessarabicum* (2n = 2x = 14, genome JJ), which were collinear to chromosome 4E [12, 15, 16]. Until now, some upstream genes in the anthocyanin biosynthetic pathway, such as those coding for chalcone synthase, dihydroflavonol 4-reductase and flavonoid 3′5′hydroxylase, have been cloned in wheat [17, 18]. However, correlations between these genes’ expression patterns and grain color have not been found. The molecular mechanism of blue aleurone is still unknown.

The traits related to anthocyanin biosynthesis are easily observed, and decreasing the anthocyanin content, as a type of secondary metabolite, did not impact the growth and development of plants. Special characteristics make the anthocyanin biosynthesis pathway clearer than other metabolic pathways in model plants [19]. The main structural genes of anthocyanin biosynthesis encode phenylalanine ammonia lyase, chalcone synthase, chalcone isomerase, favanone 3-hydroxylase, favonoid 3-hydroxylase, flavonoid 3', 5'-hydroxylase (F3'5'H), dihydrofavonol 4-reductase (DFR), leucoanthocyanidin dioxygenase and xavonoid 3-O-glucosyltransferase [20]. These structural genes are regulated mainly by two major classes of transcription factors: basic helix–loop–helix (bHLH) and Myb types [21]. The inactivation of any of these genes could block the whole metabolic pathway, causing a pale plant tissue phenotype. In this case, finding the inactive gene in white aleurone wheat is the key to decode the molecular mechanism of blue aleurone trait.

High-throughput RNA sequencing (RNA-Seq) is a powerful and cost-efficient tool for transcriptome analysis and transcript profiling in various plant species [22–24]. It has the advantage of providing the nucleotide sequences of genes being expressed in the transcriptome quickly. In this manuscript, RNA-Seq was employed with a focus to compare the transcripts of structural genes and transcription factors related to anthocyanin biosynthesis, in common wheat cultivars having white and blue aleurone for isolating the gene *Ba1*. Through comparison of transcript in blue aleurone and the homologous sequence on the chromosome 4 of *T*. *aestivum*, a type of bHLH transcription factor, *ThMYC4E*, was isolated and verified functionally as the candidate gene for *Ba1*.

## Materials and methods

### Plant materials

The common wheat ‘Blue 1’ and ‘Blue 2’ are addition lines derived from ‘White 1’ and ‘White 2’ with chromosome 4E from *Th*. *ponticum*, respectively. The aleurones of ‘Blue1’ and ‘White1’ were used for the RNA-Seq analysis. The near isogenic lines (NILs) ‘i:Jimai 22 blue aleurone’ were derived from ‘Jimai 22’ and ‘Zhongpulanli 1’ through six backcrosses and five inbreedings. ‘Zhongpulanli 1’ is also a blue wheat cultivar derived from *Th*. *ponticum*. In total, 72 genotypes with white aleurone were used to confirm the lack of *ThMYC4E* in *T*. *urartu*, *T*. *monococcum*, *T*. *turgidum*, *Ae*. *tauschii* or *T*. *aestivum*, and four cultivars with blue aleurone traits derived from *Th*. *ponticum* were used to verify the universality of *ThMYC4E* in the cultivars containing the 4E chromosome (S1 Table).

### Genomic DNA, total RNA and cDNA preparation

DNA was isolated from 1 g of 10-day-old seedlings according to the methods of Yan *et al*. [25]. At 14 days after anthesis, the aleurone was stripped from one grain carefully, and immediately placed into liquid nitrogen. The aleurones of 20 grains were collected for total RNA extraction. Total RNA was extracted using the Tiangen RNAprep Pure Plant Kit (Tiangen Corporation, Beijing, China) according to the standard protocol. The quality of the total RNA was checked by electrophoresis in a 1.0% agarose gel, and the concentration of total RNA was determined using a NanoDrop (Thermo Scientific, Wilmington, DE, USA). cDNA was obtained from total RNA using the Thermo RevertAid First Strand cDNA Synthesis Kit (Thermo-Fisher Scientific, Shanghai, China).

### Transcriptome analysis

The cDNA libraries of aleurone were prepared according to the manufacturer’s instructions for mRNA-Seq sample preparation (Illumina, Inc., San Diego, CA, USA). The cDNA library products were sequenced by Illumina paired-end sequencing technology with read lengths of 100bp on the Illumina HiSeq 2000 instrument by Huada Technologies Co. Ltd. (Beijing, China). The raw sequence reads were stored in the NCBI SRA database with the accession number SRP107065.

Before assembly, the raw paired-end reads were filtered to obtain high-quality clean reads. Low quality sequences were removed, including sequences with ambiguous bases (denoted with more than 5% “N” in the sequence trace) and low quality reads (the rate of reads in which a quality value ≤ 10 is greater than 20%) and reads with adaptors. After purity filtering was completed, the high-quality reads were assembled by Trinity with default parameters to construct unique consensus sequences based on the sequences from both white and blue aleurone genotype [26].

Unigenes that were differentially expressed between blue and white aleurones were analyzed using chi-square tests with IDEG6 software [27]. The unigene expression level was calculated using the fragments per kb per million reads (FPKM) values. The false discovery rate (FDR) method was introduced to determine the threshold p-value at FDR ≤ 0.001, and the absolute value of |log2Ratio| ≥ 1 was used as the threshold to determine the significance of the unigenes’ differential expression.

The genes related to anthocyanin biosynthesis in Kyoto Encyclopedia of Genes and Genomes (KEGG) pathways (http://www.genome.jp/kegg/) were collected and aligned to the unigenes from a transcriptome mixture of blue and white aleurones using the BLASTX algorithm with an E-value of < 1e-5. The unigenes related to anthocyanin biosynthesis were aligned to the chromosome based on the BLASTX algorithm with an E-value of < 1e-5 and the reference database (ta_IWGSC_MIPSv2.2_HighConf_CDS_2014Jul18.fa).

### PCR and semi-quantitative PCR

PCR amplification was conducted using high-fidelity Phushion DNA polymerase (Thermo-Fisher Scientific) in the GeneAmp PCR System 9700 (Thermo-Fisher Scientific), which employed the following procedure: 2 min of denaturation at 98°C; 35 cycles of 15 s at 98°C, 30 s at 61°C, and 60 s at 72°C; followed by a final extension of 5 min at 72°C. The PCR products were extracted from 1.0% agarose gels using the Tiangen TIAN gel Midi Purification Kit (Tiangen) and were cloned into the pGEM-T Easy Vector plasmid (Promega Corporation, Madison, WI, USA). The recombinant plasmids were then transformed into *Escherichia coli* DH5α cells, and six positive clones were sequenced by a commercial company (Huada Gene, Shenzheng, China). All of the primers used in this study are listed in S2 Table. The coding sequence of *ThMYC4E* was stored in National Center for Biotechnology Information (http://www.ncbi.nlm.nih.gov/genbank/) under accession number KX914905. In all of the semi-quantitative RT-PCR experiments conducted in this work, amplification of wheat tubulin gene transcripts was used to normalize the cDNA contents of various reverse transcription mixtures. The reproducibility of the transcriptional patterns revealed by semi-quantitative PCR was tested in at least three independent assays.

### Bioinformatics analysis

The sequence alignments were conducted using Vector NTI 10 software (Thermo-Fisher Scientific). The primers were designed using Primer5 software (Premier Biosoft, Palo Alto, CA, USA). The conservative functional domains were predicted using the web site (http://blast.ncbi.nlm.nih.gov/Blast.cgi?PROGRAM=blastp&PAGE_TYPE=BlastSearch&LINK_LOC=blasthome). The phylogenetic trees of the amino acid sequences of bHLH transcription factors were constructed using MEGA 6.0 [28].

### Transient expression by particle bombardment

The transient plasmids pBRACT214:ThMYC4E, pBRACT214:ZmR and pBRACT214:ZmC1 were constructed using the Gateway Cloning Kit (Thermo-Fisher Scientific) with vector pBRACT214 carrying the ubiquitin promoter. The plasmids were delivered into the coleoptiles of ‘Opata’ by particle bombardment according to the methods of Ahmed *et al*.[29]. All of the treated coleoptiles were observed two days after bombardment and photographed using a stereoscope (Leica Co., Oskar Barnack, Germany).

## Results

### Transcriptome analyses of blue and white aleurones from ‘Blue 1’ and ‘White 1’

After filtering, 34.59 M reads for white aleurone of ‘White 1’ and 23.48 M for blue aleurone of ‘Blue 1’ remained, with Q30 percentages of 95.12% and 78.54%, respectively. The high-quality reads were aligned to assemble 73,728 unigenes with an average length of 550 nt and an N50 length of 696 nt using Trinity software. Putative differentially expressed unigenes between blue and white aleurones were identified on the basis of FPKM values calculated from the read counts mapped onto the reference transcriptome. A total of 5,828 unigenes were differentially expressed between blue and white aleurones according to a comparison of expression levels with FDR ≤ 0.001 and |log2Ratio| ≥ 1 (S1 Fig). Using white aleurone as the reference, 4,176 up-regulated unigenes (with greater levels of expression in blue aleurones) and 1,652 down-regulated unigenes (with lower levels of expression in blue aleurones) were identified.

**Fig 1:**
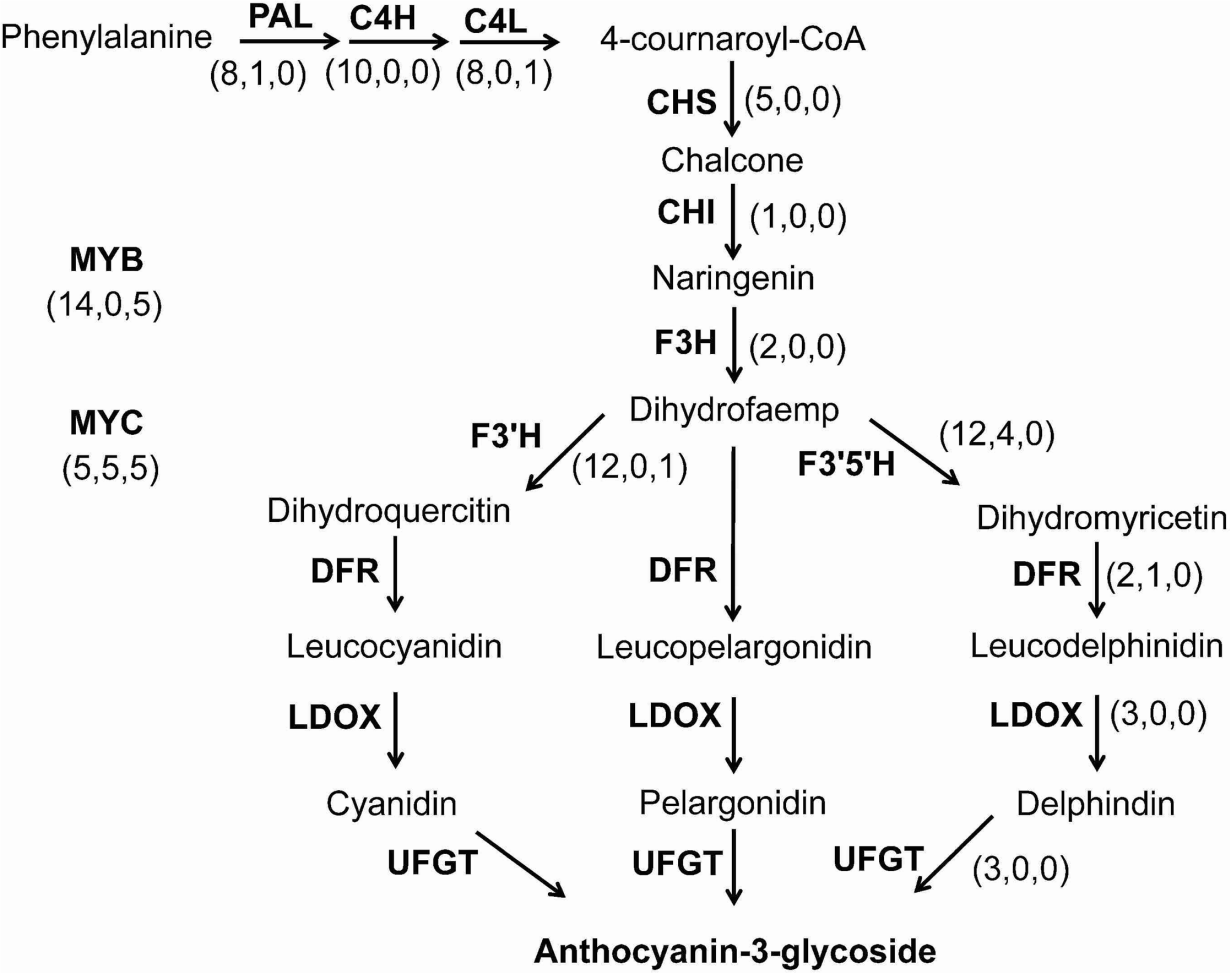
The expression differences of structural genes and transcription factors related to the anthocyanin biosynthesis pathway in blue and white aleurones. Arrows show the metabolic stream, and the abbreviations represent the genes catalyzing the progress. The first number in the parentheses represent the number of assembled unigenes, the second number represents the number of up-regulated unigenes in blue aleurone compared with white aleurone, and the third number represents the unigenes with homologous genes residing on Chromosome 4 of *Triticum aestivum*.

To further clarify the key genes responsible for the blue aleurone trait, 11 structural genes and two transcription factors (Fig 1), related to anthocyanin biosynthesis, were selected as prey for a BLAST search of the assembled unigene database. In total, 77 unigenes were homologous to those in anthocyanin biosynthesis, and only 8 unigenes had higher transcript levels in blue aleurone than in white. The eight unigenes consisted of one phenylalanine ammonia lyase (PAL), one F3′5′H, one DFR and five bHLH transcription factors (Fig 1 and S3 Table). The homologous sequences of *Ba1* have been suggested to reside on chromosome 4 of common wheat [8, 30]. The chromosomal locations of the homologues of *PAL*, *F3′5′H* and *DFR* were on chromosomes 2B, 1A and 7D, respectively, while all five of the *bHLH* transcription factors resided on chromosome 4D (S3 Table). The transcript levels of the five *bHLH* transcription factors were very low in white aleurone, with FPKM< 0.29, while the greatest FPKM value was 19.14 in blue aleurone (S3 Table), indicating that this gene might not be expressed in white aleurone. Sequence analysis revealed that the five *bHLH* transcription factors were derived from the same contig, CL3336.Contig. Based on the sequence of CL3336.Contig, a bHLH transcription factor(*ThMYC4E*) was isolated only from the cDNA of blue aleurone as the candidate *Ba1* gene. In the white aleurones, the transcript of *TaMYC4E* could not be detected (S2 Fig).

### Molecular characteristics and transcriptional function of *ThMYC4E*

bHLH gene was important for anthocyanin biosynthesis in plant. The bHLH genes *RS*, *B*, *Sn* and *Hopi* were subsequently identified in maize and shown to induce tissue-specific anthocyanin biosynthesis, including expression in the aleurone layer, scutellum, pericarp, root, mesocotyl, leaf and anther [31–35]. In white rice varieties, a 2-bp (GT) insertion in the exon 7 of the Ra gene caused frame shift mutation [36]. Homologues of the maize *R* and *B* genes were also be find in *Antirrhinum* (*Delila*) [37], petunia (*Jaf13)* [38], and tomato(*ah*) [39] influencing anthocyanin biosynthesis.

**Fig 2:**
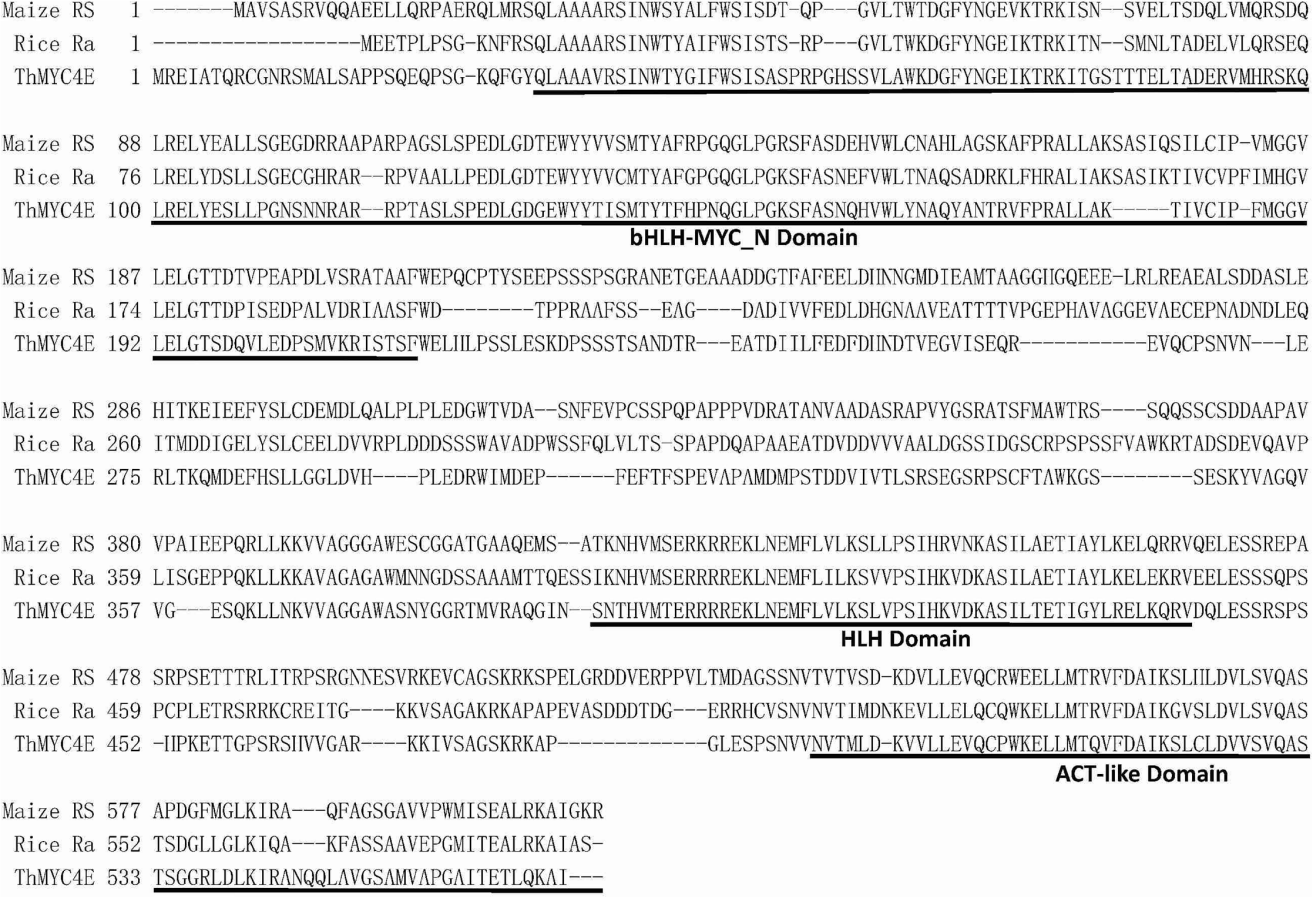
Amino acid sequence alignment of ThMYC4E and the known anthocyanin bHLH regulators RS and Ra from maize and rice, respectively. The black lines represent the conserved bHLH-MYC_N, HLH and ACT-like domains. The accession number of these proteins (ortranslated products) areas follows in the GenBank database: Rice\Ra: AAC49219, Maize/RS: NP_001106073; and ThMYC4E: KX914905.

A phylogenetic tree was constructed with the neighbor-joining method using the full-length amino acid sequences of bHLH transcription factors. ThMYC4E, encoding a protein with 586 amino acids, belonged to the branch inclucing the bHLH proteins regulating anthocyanin biosynthesis in rice, common wheat, barley, maize and sorghum (S3 Fig). bHLH proteins from the same species clustered together in the phylogenetic tree, and ThMYC4E was independent, distinguished from the bHLH proteins of maize, rice and wheat species (S3 Fig), which implied that *ThMYC4E* was not from the chromosomes of *T*. *aestivum*. For the bHLH proteins, the three domains, bHLH-MYC_N, HLH and ACT-like, are important for exercising their transcriptional functions. The bHLH-MYC_N domain is required for the protein-protein interaction with MYB transcription factors, the HLH domain facilitates DNA binding, and the ACT-like domain interacts with the RNA polymerase II machinery and then initiates transcription [35, 40]. The ThMYC4E protein contained the intact bHLH-MYC_N, HLH and ACT-like domains compared with the functional bHLH proteins RS and Ra from maize and rice, respectively (Fig 2).

**Fig 3:**
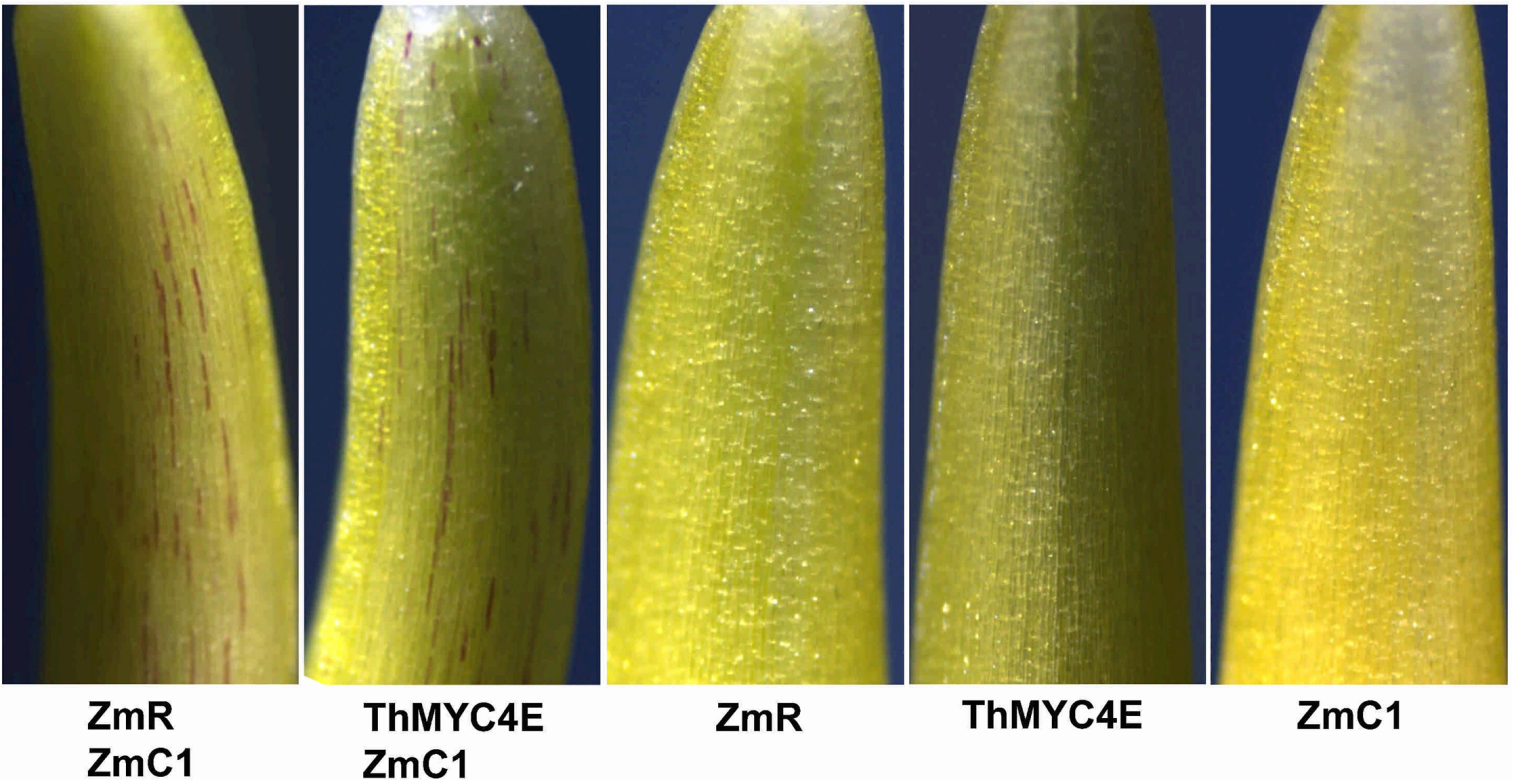
Wheat coleoptiles two days after bombardment with different plasmids. ZmC1, ZmR and ThMYC4E represent the constructs pBract214:ZmC1, pBract214:ZmR and pBract214:ThMYC4E, respectively.

The bHLH transcription factor *ZmR* induces anthocyanin biosynthesis when coexpressed with the *MYB* gene *ZmC1* [29]. In the present experiment, *ZmR* and *ZmC1* were isolated from maize to compare the function of *ThMYC4E* with that of *ZmR*. The coding sequences of *ThMYC4E*, *ZmR* and *ZmC1* were placed after the ubiquitin promoter in the pBRACT214 vector. The transient expressions of *ZmR* or *ThMYC4E* induced anthocyanin biosynthesis in the coleoptile cells of *T*. *aestivum* cv. ‘Opata’ in the presence of *ZmC1*, while *ZmR*, *ThMYC4E* or *ZmC1* alone could not independently induce anthocyanin biosynthesis (Fig 3). Thus, *ThMYC4E* should have a similar function to *ZmR* in regulating anthocyanin biosynthesis.

### The distribution of *ThMYC4E* in additional wheat lines, NILs and natural populations

**Fig 4:**
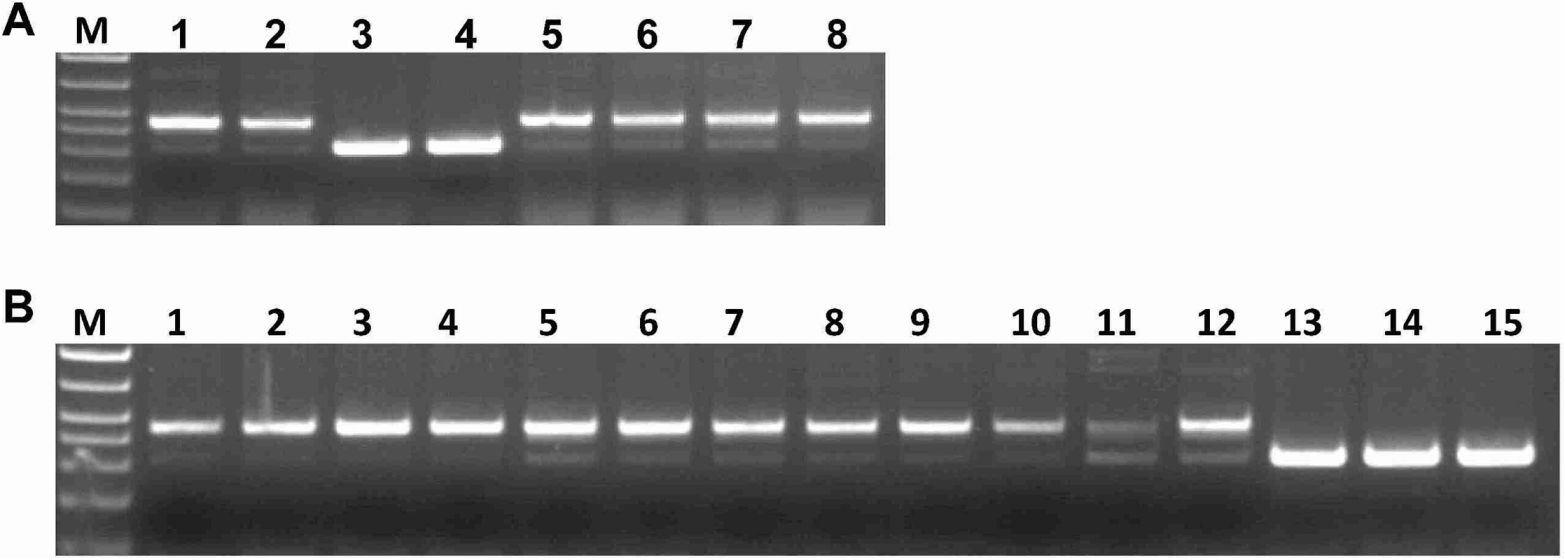
The distribution of *ThMYC4E* in addition lines, near isogenic lines and natural populations. (A) 1–8 represent ‘Blue1’, ‘Blue2’, ‘White1’, ‘White2’, ‘Blue Norco’, ‘Sebesta Blue1’, ‘Sebesta Blue 2’ and ‘Sebesta Blue 3’, respectively. (B) 1–3 represent ‘Zhongpulanli 1’, 4–12 represent ‘i:Jimai 22 blue aleurone’ near isogenic lines, and 13–15 represent three replicates of ‘Jimai 22’.

Based on the coding sequences, the primers ThMYC4Esp were designed to differentiate *ThMYC4E* from homologous genes in *T*. *aestivum*. These primers amplified products of 458bp from the genomic DNA of ‘Blue 1’ and ‘Blue 2’ carrying chromosome 4E, while the white genotypes had amplicons of smaller size as compared to blue genotypes (Fig 4A). After sequencing, the 458-bp amplification product was found to be the same as the coding sequence of ThMYC4E with an intron of 127 bp, while the amplification product from white aleurone wasn’t. The germplasms of 12 *T*. *uratu*, 3 *T*. *monococcum*, 4 *T*. *turgidum*, 10 *Ae*. *tauschii* and 43 *T*. *aestivum* genotypes were checked using the special primers (S1 Table). *ThMYC4E* was present only in blue aleurone genotypes ‘Blue Norco’, ‘Sebesta Blue1’, ‘Sebesta Blue 2’ and ‘Sebesta Blue 3’ (Fig 4A). ‘Blue Norco’ received its blue aleurone trait from *Agropyron tricophorum* [14], while those of ‘Sebesta Blue1’, ‘Sebesta Blue 2’ and ‘Sebesta Blue 3’ were derived from *Agropyron elongatum* [7]. *Agropyron elongatum* was the same species, endowing ‘Blue1’ and ‘Blue2’ the blue aleurone trait. *Agropyron tricophorum* was taxonomically very close to *Agropyron elongatum* and may contain the same chromosome 4E. *ThMYC4E* were also detected in 9 ‘i:Jimai 22 blue aleurone’ NILs and ‘Zhongpulanli 1’ (Fig 4B). However, *ThMYC4E* was not detected in the parent ‘Jimai 22’ that has white aleurone (Fig 4B). *ThMYC4E* in the NILs should be from the cultivar ‘Zhongpulanli 1’.

## Discussion

Blue colors in plant tissues can be achieved through the accumulation of delphinidin-based anthocyanins often modified with aromatic acyl groups, higher (neutral) vacuolar pH levels and the presence of co-pigments and/or metal ions, while red and pink colors usually were derived from the cyanidin-based or pelargonidin-based anthocyanins [41]. Compared with cyanidin-based and pelargonidin-based anthocyanin, the formation of delphinine needed the expression of a special structural gene *F3'5'H* (Fig 1) [42]. The structural gene *F3'5'H* had a higher transcript level in blue aleurone than white, which could imply that delphinidin-based anthocyanin was responsible for the aleurone’s blue color in ‘Blue 1’. The essential genes *PAL*, *DFR* and *ThMYC4E* for anthocyanin biosynthesis also have higher transcript level in blue aleurone, which was consistent to the higher anthocyanin content. The homologous sequences of three structural genes didn’t reside on the chromosome 4 of *T*. *aestivum*, while the transcriptor *ThMYC4E* did. The homologous sequences of *Ba1* have been suggested to reside on chromosome 4 of common wheat [4, 8]. So *ThMYC4E* has the possibility to be *Ba1*, while *F3'5'H*, *PAL* and *DFR* don’t. *ThMYC4E* had the characteristic domains, bHLH-MYC_N, HLH and ACT-like, of a bHLH gene and a high similarity to RS and Ra [35, 36], which regulate anthocyanin biosynthesis in maize and rice, respectively. The transient expression of *ThMYC4E* could induce anthocyanin biosynthesis in the coleoptiles of ‘Opata’ with the help of a MYB transcriptor *ZmC1*. The isolation of a functional bHLH transcriptor confirmed the proposal that RNA-Seq is a powerful method to study gene expression for any gene of interest [43].

In the phylogenetic tree composed of bHLH proteins regulating anthocyanin biosynthesis, *ThMYC4E* was distinguished from the bHLH proteins from *T*. *uratu*, *Ae*. *tauschii* and *T*. *aestivum*. ‘Sebesta Blue 1’, ‘Sebesta Blue 2’ ‘Blue 1’, ‘Blue 2’ and ‘Blue Norco’ have disomic additions of chromosome 4E [6, 7, 44], while ‘Sebesta Blue 3’ carry the segment of chromosome 4E [6, 7]. *ThMYC4E* existed in these cultivars, but did not exist in *T*. *uratu*, *T*. *monococcum*, *T*. *turgidum*, *Ae*. *tauschii*or, and *T*. *aestivum*. Compared with common wheat, the whole or segment of chromosome 4E should be the only unique chromosome in ‘Blue 1’, ‘Blue 2’, ‘Blue Norco’, ‘Sebesta Blue 1’, ‘Sebesta Blue 2’, and ‘Sebesta Blue 3’, which implies that *ThMYC4E* originally resided on chromosome 4E.

bHLH transcription factors play important roles in activating the anthocyanin biosynthesis pathway, and the functional loss of a bHLH transcription factor could induce pale traits [31–39]. The transcriptome analysis found that *ThMYC4E* was only a bHLH transcription factor with high expression in blue aleurone (Fig 1), and the transient experiment proved *ThMYC4E* had the capability of regulating anthocyanin biosynthesis as a bHLH-type transcription factor. Considering these conditions, it could be speculated that *ThMYC4E* play an important role in the anthocyanin biosynthesis of blue aleurone. After six backcrosses and five inbreedings, the NILs ‘i:Jimai 22 blue aleurone’ should theoretically have the same genetic background as ‘Jimai 22’, except *Ba1*. *Ba1* has been speculated to be controlled by a single dominant gene [6, 8], while *ThMYC4E* was the difference between ‘i:Jimai 22 blue aleurone’ and ‘Jimai 22’. It should be suggested that *ThMYC4E* was the candidate gene *Ba1* controlling blue aleurone trait.

In summary, we isolated a functional bHLH transcriptor *ThMYC4E* from the cultivar with blue aleurone trait derived from *Th*. *ponticum* through transcriptome analysis. All cultivars with blue aleurone trait derived from *Th*. *ponticum* carried this gene, implied that *ThMYC4E* was the candidate *Ba1* gene which controlled the associated trait. The *ThMYC4E* isolation should benefit to explore the molecular mechanism of the blue aleurone trait derived from alien chromosomes in *T*. *aestivum*, and to breed blue grain wheat cultivars.

## Supporting information

S1 Fig Differentially expressed genes between blue and white aleurone. The genes were classified into three classes. Red genes are up-regulated if gene expression of right sample is larger than left sample. Blue genes are down-regulated that gene expression of left sample is larger if right sample. Dark genes are not differentially expressed. The horizontal coordinates is the expression level of right and the vertical coordinates is the expression level of left sample.
(PDF)

S2 Fig The relative transcript levels of *ThMYC4E* in the aleurones of ‘Blue 1’, ‘Blue 2’, ‘White 1’ and ‘White 2’. (PDF)

S3 Fig Phylogenetic relationships between ThMYC4E and anthocyanin-related bHLHs in other species. The tree was constructed using MEGA6, neighboring-joining phylogeny testing, and 1,000 boot strap replicates. The accession number of these proteins (ortranslated products) areas follows in the GenBank database: Rice\Ra: AAC49219; Rice\R-Sx2: XP_006653664; Triticum urartu\R-S: KD049651.1; Aegilops tauschii\RS: KD512407.1; Barley\R accession AK361387.1; Triticum urartu\RS: KD032825.1; Aegilops tauschii\R-S: KD566857.1; Maize\R-S like: XP_008669036; Maize\B1: KC771884.1; Maize\CP1: NP_001105706; Sorghum\b1-1: AY542311.1; Maize/RS: NP_001106073; Maize\Hopi: CAB92300; Maize\LC: NP_001105339; Maize\SN: NP_001105339; Maize\r1-B3: NP_001105339; Arabidopsis\GL3: NP_680372; Arabidopsis\EGL3: NP_176552; petunia\AF13: AAC39455; Arabidopsis\MYC1: NP_191957; Tobacco\AN1a: AEE99257; Tobacco\AN1-like: NM_001302566.1; Tobacco\AN1b: HQ589209.1; Petunia\AN1: AF260918.1; Medicago\TT8: AF260918.1; Arabidopsis\TT8: CAC14865; Maize\IN1: AAB03841; ThMYC4E: KX914905.
(PDF)

S1 Table The origin, phenotype and genotype of materials used in this study. (PDF)

S2 Table Names and sequences of the primers used in this study. (PDF)

S3 Table The information of unigenes relative to anthocyanin biosynthesis in aleurones. (PDF)

## References

[1] Abdel-Aal ESM, Hucl P. Composition and stability of anthocyanins in blue-grained wheat. J Agri Food Chem. 2003;51(8): 2174–2180. 10.1021/jf021043x 12670152

[2] Trojan V, Musilová M, Vyhnánek T, Klejdus B, Hanáček P, Havel L. Chalcone synthase expression and pigments deposition in wheat with purple and blue colored caryopsis. J Cereal Sci. 2004;59(1): 48–55.

[3] Knievel DC, Esm AA, Rabalski I, Nakamura T, Hucl P. Grain color development and the inheritance of high anthocyanin blue aleurone and purple pericarp in spring wheat (*Triticum aestivum L*.). J Cereal Sci. 2009;50(1): 113–120.

[4] Zeven AC. Wheats with purple and blue grains: a review. Euphytica. 1991;56(3): 243–258.

[5] Hucl P, Matus-Cádiz M. Isolation distances for minimizing out-crossing in spring wheat. Crop Sci. 2001;41(4): 1348–1351.

[6] Metzger RJ, Sebesta E. Registration of three blue-seeded wheat genetic stocks exhibiting xenia. Crop Sci. 2004;44(6): 2281–2282.

[7] Morrison LA, Metzger RJ, Lukaszewski AJ. Origin of the blue-aleurone gene in Sebesta Blue wheat genetic stocks and a protocol for its use in apomixis screening. Crop Sci. 2004 44(6): 2063–2067.

[8] Zheng Q, Li B, Zhang XY, Mu SM, Zhou HP, Li ZS. Molecular cytogenetic characterization of wheat-*Thinopyrum ponticum* translocations bearing blue-grained gene(s) induced by r-ray. Euphytica. 2006;152(1): 51–60.

[9] Zhou K, Wang S, Feng Y, Liu Z, Wang G. The 4E- system of producing hybrid wheat. Crop Sci. 2006;46(1): 250–255.

[10] Wang LS, Stoner GD. Anthocyanins and their role in cancer prevention. Cancer Lett. 2008;269(2): 281–290. doi: 10.1016/j.canlet.2008.05.020 18571839 10.1016/j.canlet.2008.05.020 PMC2582525

[11] Bowen-Forbes CS, Zhang YJ, Nair MG. Anthocyanin content, antioxidant, anti-inflammatory and anticancer properties of blackberry and raspberry fruits. J Food Compos Anal. 2010;23(6): 554–560.

[12] Zeller FJ, Cermeño MC, Miller TE Cytological analysis on the distribution and origin of the alien chromosome pair conferring blue aleurone color in several European common wheat (*Triticum aestivum L*.) strains. Theor Appl Genet. 1991;81(4): 551–558. doi: 10.1007/BF00219448 24221323 10.1007/BF00219448

[13] Li ZS, Mu SM, Zhou HP, Wu JK. The establishment and application of blue-grained monosomics in wheat chromosome engineering. Cereal Res Commun. 1986;14(2): 133–137

[14] Whelan ED. Transmission of an alien telocentric addition chromosome in common wheat that confers blue seed color. Genome. 2011;32(1): 30–34.

[15] Dubcovsky J, Luo MC, Zhong GY, Bransteitter R, Desai A, Kilian A, et al Genetic map of diploid wheat, *Triticum monococcum L*., and its comparison with maps of *Hordeum vulgare L*. Genetics. 1996;143(2): 983–999. 8725244 10.1093/genetics/143.2.983 PMC1207354

[16] Singh K, Ghai M, Garg M, Chhuneja P, Kaur P, Schnurbusch T, et al An integrated molecular linkage map of diploid wheat based on a *Triticum boeoticum* x *T*. *monococcum* RIL population. Theor Appl Genet. 2007;115(3):301–312. doi: 10.1007/s00122-007-0543-z 17565482 10.1007/s00122-007-0543-z

[17] Yang GH, Zhao XQ, Li B, Liu JZ, Zheng Q, Tong YP, et al Molecular cloning and characterization of a DFR from developing seeds of blue-grained wheat in anthocyanin biosynthetic pathway. Act Bot Sin. 2003;45(11): 1329–1338.

[18] Yang GH, Li B, Gao J, Liu J, Zhao XQ, Zheng Q, et al Cloning and expression of two chalcone synthase and a flavonoid 3′5′—hydroxylase 3′-end cDNAs from developing seeds of blue—grained wheat involved in anthocyanin biosynthetic pathway. Acta Bot Sin. 2004;46(5): 588–594.

[19] Saito K, Yonekura-Sakakibara K, Nakabayashi R, Higashi Y, Yamazaki M, Tohge T, et al The flavonoid biosynthetic pathway in *Arabidopsis*: structural and genetic diversity. Plant Physiol Bioch. 2013;72: 21–34. 10.1016/j.plaphy.2013.02.001 23473981

[20] Zhang Y, Butelli E, Martin C. Engineering anthocyanin biosynthesis in plants. Curr Opin Plant Biol. 2014;19:81–90. doi: 10.1016/j.pbi.2014.05.011 24907528 10.1016/j.pbi.2014.05.011

[21] Xu W, Dubos C, Lepiniec L. Transcriptional control of flavonoid biosynthesis by MYB–bHLH–WDR complexes. Trends Plant Sci. 2015;20(3): 176–185. doi: 10.1016/j.tplants.2014.12.001 25577424 10.1016/j.tplants.2014.12.001

[22] Dorn KM, Fankhauser JD, Wyse DL, Marks MD. De novo assembly of the pennycress (*Thlaspi arvense*) transcriptome provides tools for the development of a winter cover crop and biodiesel feedstock. Plant J. 2013;75(6): 1028–1038. doi: 10.1111/tpj.12267 23786378 10.1111/tpj.12267 PMC3824206

[23] Firon N, LaBonte D, Villordon A, Kfir Y, Solis J, Lapis E, et al Transcriptional profiling of sweetpotato (*Ipomoea batatas*) roots indicates down-regulation of lignin biosynthesis and up-regulation of starch biosynthesis at an early stage of storage root formation. BMC Genomics. 2013;14(1): 460. 23834507 10.1186/1471-2164-14-460 PMC3716973

[24] Zhang N, Liu B, Ma C, Zhang G, Chang J, Si H, et al. Transcriptome characterization and sequencing-based identification of drought-responsive genes in potato. Mol Biol Rep. 2014;41(1): 505–517. doi: 10.1007/s11033-013-2886-7 24293150 10.1007/s11033-013-2886-7

[25] Yan ZH, Wan YF, Liu KF, Zheng YL, Wang DW. Identification of a novel HMW glutenin subunit and comparison of its amino acid sequence with those of homologous subunits. Chin Sci Bull. 2002;47(3): 222–227.

[26] Grabherr MG, Haas BJ, Yassour M, Levin JZ, Thompson DA, Amit I, et al Trinity: reconstructing a full-length transcriptome without a genome from RNA-Seq data. Nat Biotechnol. 2013;29(7): 644–652. doi: 10.1038/nbt.1883 10.1038/nbt.1883 PMC3571712 21572440

[27] Romualdi C, Bortoluzzi S, d’Alessi F, Danieli GA. IDEG6: a web tool for detection of differentially expressed genes in multiple tag sampling experiments. Physiol Genomics 2003;12(2):159–162. doi: 10.1152/physiolgenomics.00096.2002 12429865 10.1152/physiolgenomics.00096.2002

[28] Tamura K, Dudley J, Nei M, Kumar S. MEGA4: Molecular evolutionary genetics analysis (MEGA) software version 4.0. Mol Biol Evol. 2007;24(8):1596–1599. doi: 10.1093/molbev/msm092 17488738 10.1093/molbev/msm092

[29] Ahmed N, Maekawa M, Utsugi S, Himi E, Ablet H, Rikiishi K, et al. Transient expression of anthocyanin in developing wheat coleoptile by maize *C1* and *B-peru* regulatory genes for anthocyanin synthesis. Breeding Sci. 2003;53(1): 29–34.

[30] Zheng Q, Li B, Li HW, Li ZS. Utilization of blue-grained character in wheat breeding derived from *Thinopyrum poticum*. J Genet Genomics. 2009;36(9): 575–580. doi: 10.1016/S1673-8527(08)60149-6 19782959 10.1016/S1673-8527(08)60149-6

[31] Tonelli C, Consonni G, Dolfini SF, Dellaporta SL, Viotti A, Gavazzi G. Genetic and molecular analysis of Sn, a light-Inducible, tissue specific regulatory gene in maize. Mol Gen Genet. 1991;225(3): 401–410. 1673220 10.1007/BF00261680

[32] Goff SA, Cone KC, Chandler VL Functional-analysis of the transcriptional activator encoded by the Maize-B gene—evidence for a direct functional interaction between 2 classes of regulatory proteins. Gene Dev. 1992;6(5): 864–875. 1577278 10.1101/gad.6.5.864

[33] Procissi A, Dolfini S, Ronchi A, Tonelli C. Light-dependent spatial and temporal expression of pigment regulatory genes in developing maize seeds. Plant Cell. 1997;9(9): 1547–1557. doi: 10.1105/tpc.9.9.1547 12237395 10.1105/tpc.9.9.1547 PMC157032

[34] Petroni K, Cominelli E, Consonni G, Gusmaroli G, Gavazzi G, Tonelli C. The developmental expression of the maize regulatory gene Hopi determines germination-dependent anthocyanin accumulation. Genetics. 2000;155(1): 323–336. 10790406 10.1093/genetics/155.1.323 PMC1461070

[35] Styles ED, Ceska O, Seah KT. Developmental differences in action of R and B alleles in maize. Genome. 2011;15(1): 59–72.

[36] Wang C, Shu Q. Fine mapping and candidate gene analysis of purple pericarp gene Pb in rice (*Oryza sativa L*.). Chin Sci Bull. 2007;52(22): 3097–3104.

[37] Carpenter R, Coen ES. Floral homeotic and pigment mutations produced by transposon-mutagenesis in Antirrhinum-Majus. Genes Dev. 1990;4(9): 537–544. 10.1101/gad.4.9.1483 1979295

[38] Quattrocchio F, Wing JF, Van DWK, Mol JN, Koes R. Analysis of bHLH and MYB domain proteins: species-specific regulatory differences are caused by divergent evolution of target anthocyanin genes. Plant J. 1998;13(4): 475–488. 9680994 10.1046/j.1365-313x.1998.00046.x

[39] Qiu Z, Wang X, Gao J, Guo Y, Huang Z, Du Y. The tomato Hoffman’s anthocyaninless gene encodes a bHLH transcription factor involved in anthocyanin biosynthesis that is developmentally regulated and induced by low temperatures. PLoS One 2016; 11(3): e0151067 doi: 10.1371/journal.pone.0151067 26943362 10.1371/journal.pone.0151067 PMC4778906

[40] Atchley WR, Wollenberg KR, Fitch WM, Terhalle W, Dress AW. Correlations among amino acid sites in bHLH protein domains: an information theoretic analysis. Mol Bio Evol. 2000;17(1): 164–178. 10666716 10.1093/oxfordjournals.molbev.a026229

[41] Mori M, Kondo T, Yoshida K. Anthocyanin components and mechanism for color development in blue Veronica flowers. Biosci Biotechnol Biochem. 2009;73(10): 2329–2331. 19809174 10.1271/bbb.90349

[42] Whang SS, Wan SU, Song IJ, Lim PO, Choi K, Park KW, et al Molecular analysis of anthocyanin biosynthetic genes and control of flower coloration by flavonoid 3’,5’-hydroxylase (F3’5’H) in *dendrobium moniliforme*. J Plant Biol. 2011;54(3): 209–218.

[43] Liu D, Li SM, Chen WJ, Zhang B, Liu DC, Liu BL et al Transcriptome analysis of purple pericarps in common wheat (*Triticum aestivumL*.). PLoS One. 2016;11(5): e0155428 doi: 10.1371/journal.pone.0155428 27171148 10.1371/journal.pone.0155428 PMC4865117

[44] Buresova V, Kopecky D, Bartos J, Martinek P, Watanabe N, Vyhnanek T, et al Variation in genome composition of blue-aleurone wheat. Theor Appl Genet. 2015;128(2): 273–282. doi: 10.1007/s00122-014-2427-3 25399318 10.1007/s00122-014-2427-3

