# Supplementary material for: ThMYC4E, candidate Blue aleurone 1 gene controlling the associated trait in Triticum aestivum
Source: PLoS One. 2017 Jul 13;12(7):e0181116. doi: 10.1371/journal.pone.0181116 (PMC5509306; doi:10.1371/journal.pone.0181116)
Supplement: S1 Fig — The genes were classified into three classes. Red genes are up-regulated if gene expression of right sample is larger than left sample. Blue genes are down-regulated that gene expression of left sample is larger if right sample. Dark genes are not differentially expressed. The horizontal coordinates is the expression level of right and the vertical coordinates is the expression level of left sample. (PDF) [file pone.0181116.s001.pdf]

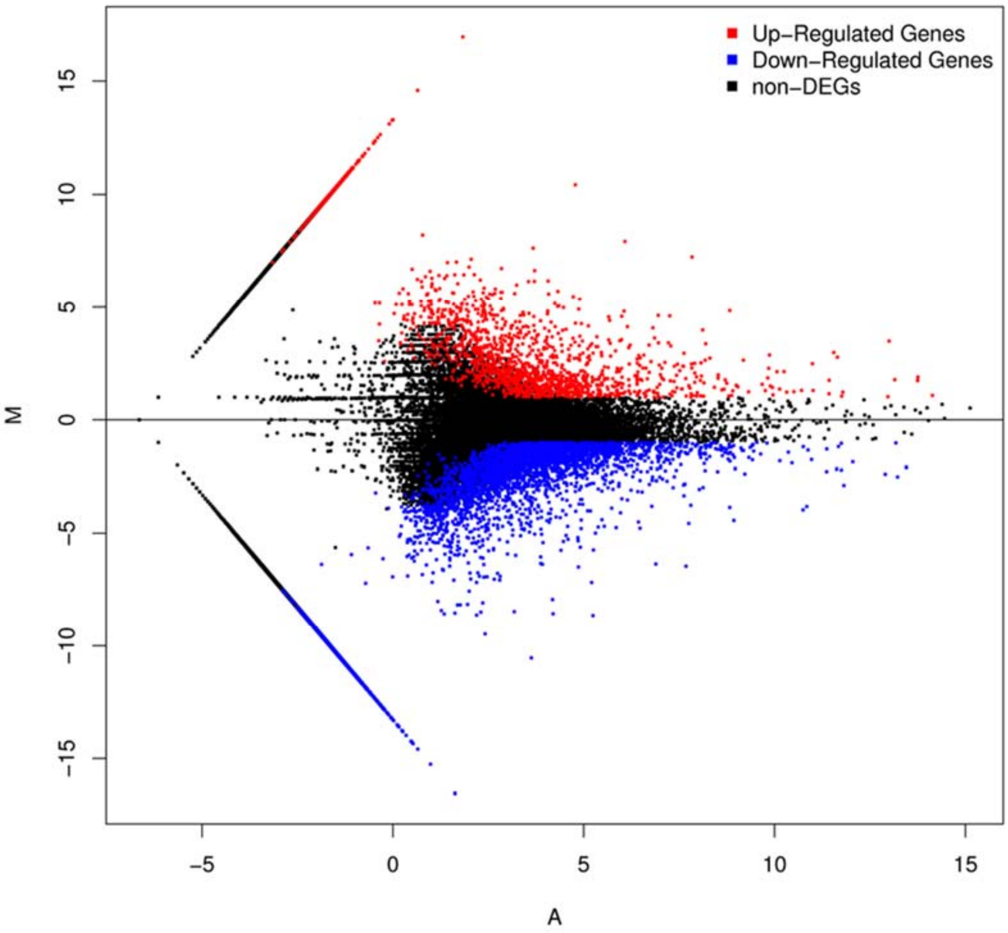

S1 Fig. Differentially expressed genes between blue and white aleurone. The genes were classified into three classes. Red genes are up-regulated if gene expression of right sample is larger than left sample. Blue genes are down-regulated that gene expression of left sample is larger if right sample. Dark genes are not differentially expressed. The horizontal coordinates is the expression level of right and the vertical coordinates is the expression level of left sample.
