# Supplementary figures and images for: ThMYC4E, candidate Blue aleurone 1 gene controlling the associated trait in Triticum aestivum
Source: PLoS One. 2017 Jul 13;12(7):e0181116. doi: 10.1371/journal.pone.0181116 (PMC5509306; doi:10.1371/journal.pone.0181116)

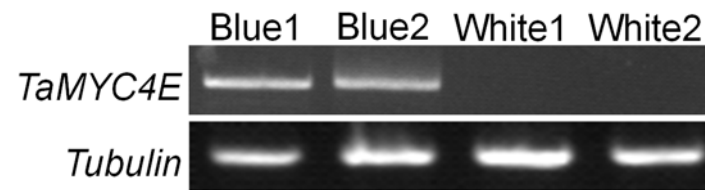

**S2 Fig.** The relative transcript levels of *ThMYC4E* in the aleurones of ‘Blue 1’, ‘Blue 2’, ‘White 1’ and ‘White 2’.

Supplement: S2 Fig — (PDF) [file pone.0181116.s002.pdf]
