# Supplementary material for: ThMYC4E, candidate Blue aleurone 1 gene controlling the associated trait in Triticum aestivum
Source: PLoS One. 2017 Jul 13;12(7):e0181116. doi: 10.1371/journal.pone.0181116 (PMC5509306; doi:10.1371/journal.pone.0181116)
Supplement: S3 Fig — The tree was constructed using MEGA6, neighboring-joining phylogeny testing, and 1,000 boot strap replicates. The accession number of these proteins (ortranslated products) areas follows in the GenBank database: Rice\Ra: AAC49219; Rice\R-Sx2: XP_006653664; Triticum urartu\R-S: KD049651.1; Aegilops tauschii\RS: KD512407.1; Barley\R accession AK361387.1; Triticum urartu\RS: KD032825.1; Aegilops tauschii\R-S: KD566857.1; Maize\R-S like: XP_008669036; Maize\B1: KC771884.1; Maize\CP1: NP_001105706; Sorghum\b1-1: AY542311.1; Maize/RS: NP_001106073; Maize\Hopi: CAB92300; Maize\LC: NP_001105339; Maize\SN: NP_001105339; Maize\r1-B3: NP_001105339; Arabidopsis\GL3: NP_680372; Arabidopsis\EGL3: NP_176552; petunia\AF13: AAC39455; Arabidopsis\MYC1: NP_191957; Tobacco\AN1a: AEE99257; Tobacco\AN1-like: NM_001302566.1; Tobacco\AN1b: HQ589209.1; Petunia\AN1: AF260918.1; Medicago\TT8: AF260918.1; Arabidopsis\TT8: CAC14865; Maize\IN1: AAB03841; ThMYC4E: KX914905. (PDF) [file pone.0181116.s003.pdf]

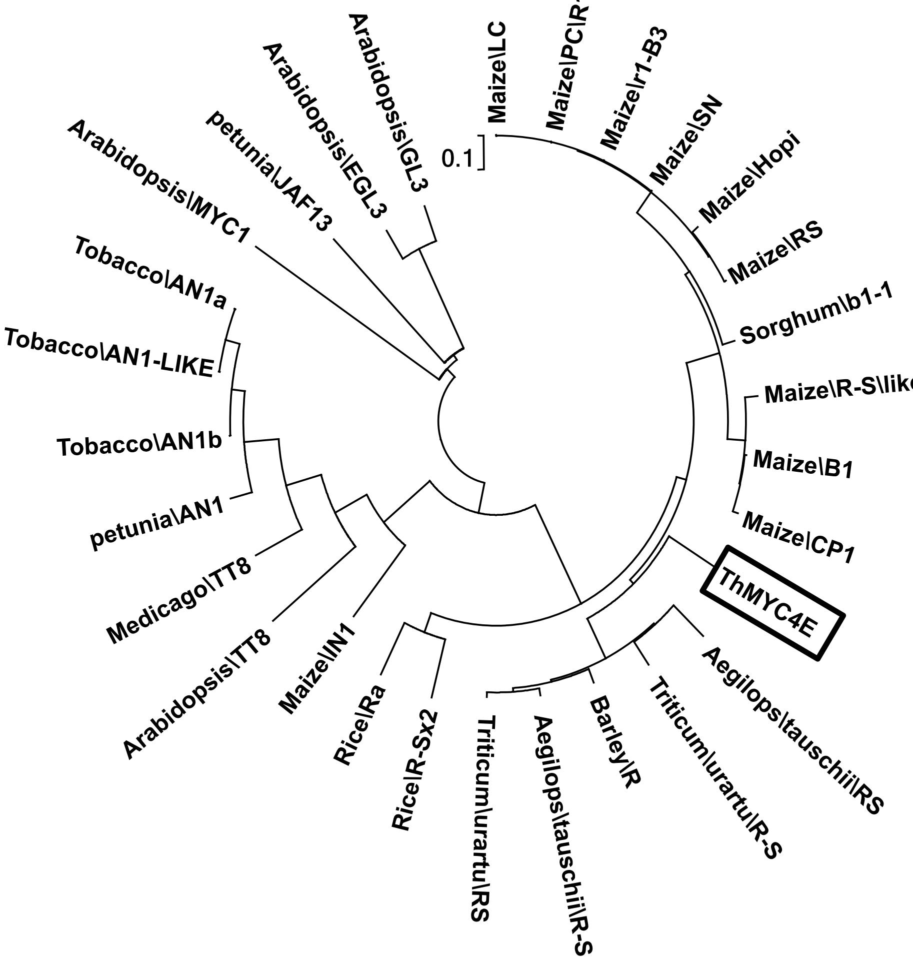

S3 Fig. Phylogenetic relationships between ThMYC4E and anthocyanin-related bHLHs in other species. The tree was constructed using MEGA6, neighboring-joining phylogeny testing, and 1,000 boot strap replicates. The accession number of these proteins (ortranslated products) areas follows in the GenBank database: Rice\Ra: AAC49219; Rice\R-Sx2: XP_006653664; Triticum urartu\R-S: KD049651.1; Aegilops tauschii\RS: KD512407.1; Barley\R accession AK361387.1; Triticum urartu\RS: KD032825.1; Aegilops tauschii\R-S: KD566857.1; Maize\R-S like: XP_008669036; Maize\B1: KC771884.1; Maize\CP1: NP_001105706; Sorghum\b1-1: AY542311.1; Maize/RS: NP_001106073; Maize\Hopi: CAB92300; Maize\LC: NP_001105339;

Maize\SN: NP_001105339; Maize\r1-B3: NP_001105339; Arabidopsis\GL3: NP_680372; Arabidopsis\EGL3: NP_176552; petunia\AF13: AAC39455; Arabidopsis\MYC1: NP_191957; Tobacco\AN1a: AEE99257; Tobacco\AN1-like: NM_001302566.1; Tobacco\AN1b: HQ589209.1; Petunia\AN1: AF260918.1; Medicago\TT8: AF260918.1; Arabidopsis\TT8: CAC14865; Maize\IN1: AAB03841; ThMYC4E: KX914905.
