# Supplementary material for: ThMYC4E, candidate Blue aleurone 1 gene controlling the associated trait in Triticum aestivum
Source: PLoS One. 2017 Jul 13;12(7):e0181116. doi: 10.1371/journal.pone.0181116 (PMC5509306; doi:10.1371/journal.pone.0181116)
Supplement: S1 Table — (PDF) [file pone.0181116.s004.pdf]

S1 Table. The origin, phenotype and genotype of materials used in this study.

Item	Taxon	Plant ID	Name	Country	Phenotype	*ThMYC4E*
1	Triticum uratu	Cltr17664	G3135	Lebanon,EI Beqaa	White	-
2	Triticum uratu	Cltr17666	G3162	Lebanon,EI Beqaa	White	-
3	Triticum uratu	Cltr17667	G1785	Turkey,mardin	White	-
4	Triticum uratu	PI428181	G1753	Armenia	White	-
5	Triticum uratu	PI428184	G1785	Turkey,Mardin	White	-
6	Triticum uratu	PI428328	G3254	Lebanon,EI Beqaa	White	-
7	Triticum uratu	PI428329	G3255	Lebanon,EI Beqaa	White	-
8	Triticum uratu	PI428330	G3256	Lebanon,EI Beqaa	White	-
9	Triticum uratu	PI428332	G3259	Lebanon,EI Beqaa	White	-
10	Triticum uratu	PI428333	G3261	Lebanon,EI Beqaa	White	-
11	Triticum uratu	PI428334	G3262	Lebanon,EI Beqaa	White	-
12	Triticum uratu	PI428336	G3264	Lebanon,EI Beqaa	White	-
13	Triticum uratu	PI428339	G3269	Lebanon,EI Beqaa	White	-
14	Triticum uratu	PI487269	SY20123	Syria,As Suwayda'	White	-
15	Triticum uratu	PI487270	SY20125	Syria,Dimashq	White	-
16	Triticum uratu	PI487271	SY20177	Syria,Idlib	White	-
17	Triticum monococcum	PI470726	79TK016-072	Turkey,Elazig	White	-
18	Triticum monococcum	PI401414	D-540	Iran, Lorestan	Blue	-
19	Triticum monococcum	PI427741	G2420	Irap,Dahuk	White	-
20	Triticum turgidum subsp.durum	AS2296			White	-
21	Triticum turgidum subsp.durum	AS2378			White	-
22	Triticum turgidum subsp.durum	AS2331			White	-
23	Triticum turgidum subsp.dicoccon	PI94655	ELS 6404-115-2	Bulgaria	White	-
24	Aegilops tauschii	CIae4	2038	Afghanistan,Ghazni	White	-
25	Aegilops tauschii	PI511363	KU-2059	Afghanistan,Faryab	White	-
26	Aegilops tauschii	PI511370	KU-2083	Iran,Mazandaran	White	-
27	Aegilops tauschii	PI554321	84TK562-005	Turkey, Hakkari	White	-
28	Aegilops tauschii	PI603220	TA1578	West Asia	White	-
29	Aegilops tauschii	PI603221	TA1597	West Asia	White	-
30	Aegilops tauschii	PI603230	TA1662	Azerbaijan	White	-
31	Aegilops tauschii	PI603234	TA1670	Azerbaijan	White	-
32	Aegilops tauschii	PI603235	TA1671	Azerbaijan	White	-
33	Aegilops tauschii	PI603251	TA2378	Iran,Gilan	White	-
34	Triticum aestivum	PI537976	24丨6	Turkey,Urfa	White	-
35	Triticum aestivum		Lemai 5	China, Qinghai	White	-
36	Triticum aestivum		Humai 13	China, Qinghai	White	-
37	Triticum aestivum		Gy2015	China, Qinghai	White	-
38	Triticum aestivum		Gy314	China, Qinghai	White	-

39	Triticum aestivum		Lantian 3	China, Qinghai	White	-
40	Triticum aestivum		Yangmai 15	China, Qinghai	White	-
41	Triticum aestivum		Gy913	China, Qinghai	White	-
42	Triticum aestivum		4211-000-30	China, Qinghai	White	-
43	Triticum aestivum		Qingchun 144	China, Qinghai	White	-
44	Triticum aestivum		GyV028	China, Qinghai	White	-
45	Triticum aestivum		Gy671	China, Qinghai	White	-
46	Triticum aestivum		Gy115	China, Qinghai	White	-
47	Triticum aestivum		Gy437	China, Qinghai	White	-
48	Triticum aestivum		GY412	China, Qinghai	White	-
49	Triticum aestivum		Qingchun 533	China, Qinghai	White	-
50	Triticum aestivum		Gy356	China, Qinghai	White	-
51	Triticum aestivum		Gy182	China, Qinghai	White	-
52	Triticum aestivum		Hanhai304	China, Qinghai	White	-
53	Triticum aestivum		Qingchun 254	China, Qinghai	White	-
54	Triticum aestivum		Gy338	China, Qinghai	White	-
55	Triticum aestivum		Gy506	China, Qinghai	White	-
56	Triticum aestivum		Humai 12	China, Qinghai	White	-
57	Triticum aestivum		Mobo	China, Qinghai	White	-
58	Triticum aestivum		Humai 14	China, Qinghai	White	-
59	Triticum aestivum		Qingchun 570	China, Qinghai	White	-
60	Triticum aestivum		Ningmai 9	China, Qinghai	White	-
61	Triticum aestivum		Qingnong 524	China, Qinghai	White	-
62	Triticum aestivum		Qingchun 37	China, Qinghai	White	-
63	Triticum aestivum		Qingchun415	China, Qinghai	White	-
64	Triticum aestivum		Qingchun 952	China, Qinghai	White	-
65	Triticum aestivum		Qingchun 38	China, Qinghai	White	-
66	Triticum aestivum		Chaichun 236	China, Qinghai	White	-
67	Triticum aestivum		Shanhan 901	China, Qinghai	White	-
68	Triticum aestivum		Gy584	China, Qinghai	White	-
69	Triticum aestivum		Bobwhite	Mexico	White	-
70	Triticum aestivum		Humai 15	China, Qinghai	White	-
71	Triticum aestivum	PI542500	R84-2794	United States,Oregon	White	-
72	Triticum aestivum	PI542501	R84-2776	United States,Oregon	White	-
73	Triticum aestivum	PI634539	Sebesta Blue-2	United States, Oregon	Blue	+
74	Triticum aestivum	PI542465	Blue Norco	United States, Oregon	Blue	+
75	Triticum aestivum	PI634538	Sebesta Blue-1	United States, Oregon	Blue	+
76	Triticum aestivum	PI634540	Sebesta Blue-3	United States, Oregon	Blue	+

Note: CIae, CItr and PI codes refer to the materials from USDA-ARS, NSGC (https://npgsweb.ars-grin.gov/gringlobal/search.aspx?), AS codes refer to the materials

from Triticeae Research Institute, Sichuan Agricultural University, and blank codes refer to the materials from Northwest Institute of Plateau Biology, Chinese Academy of Science. “+” represent the presence of *ThMYC4E* in the seventh column, and “-” represent the missing of *ThMYC4E*.
