# Supplementary material for: ThMYC4E, candidate Blue aleurone 1 gene controlling the associated trait in Triticum aestivum
Source: PLoS One. 2017 Jul 13;12(7):e0181116. doi: 10.1371/journal.pone.0181116 (PMC5509306; doi:10.1371/journal.pone.0181116)
Supplement: S2 Table — (PDF) [file pone.0181116.s005.pdf]

S2 Table. Names and sequences of the primers used in this study.

Number	Primer	Sequence (5'-3')
1	ThMYC4EcdsF	ATGCGGGAAATAGCTACTCAG
2	ThMYC4EcdsR	CTATATAGCTTTCTGAAGTGTTTCG
3	ThMYC4ESpF	CTCCCAGTCAGGAACAGC
4	TaMYC4SpR	GGTGACAGTGAGGCGGTT
5	Tubulin-F	TGAGGACTGGTGCTTACCGC
6	Tubulin-R	GCACCATCAAACCTCAGGGA
9	TaMYC1AttB1F	AAAAAGCAGGCTTC ATGCGGGAAATAGCTACTCAG
10	TaMYC1AttB2R	AAAAAGCAGGCT CTATATAGCTTTCTGAAGTGTTTCG
11	ZmRAttB1F	AAAAAGCAGGCTTCATGGCGCTTTCAGCTTCCCGAG
12	ZmRAttB2R	AGAAAGCTGGGTCTCACCGCTTCCCTATAGCTTTGC
13	ZmC1AttB1F	AAAAAGCAGGCTTCATGGGGAGGAGGGCGTGTTG
14	ZmC1AttB2R	AGAAAGCTGGGTCCTACGCAAGCTGCCCGGCCGT
15	attB1 adapter	GGGGACAAGTTTGTACAAAAAAGCAGGCT
16	attB2 adapter	GGGGACCACTTTGTACAAGAAAGCTGGGT
