# Supplementary material for: ThMYC4E, candidate Blue aleurone 1 gene controlling the associated trait in Triticum aestivum
Source: PLoS One. 2017 Jul 13;12(7):e0181116. doi: 10.1371/journal.pone.0181116 (PMC5509306; doi:10.1371/journal.pone.0181116)
Supplement: S3 Table — (PDF) [file pone.0181116.s006.pdf]

S3 Table. The information of unigenes relative to anthocyanin biosynthesis in aleurones.

Gene	KO	GeneID	Chromosome location	Length	white-Expression	blue-Expression	log^2FoldChange (blue/white)	Pvalue	FDR	Up/Down-Regulation (blue/white)
PAL	K10775	CL957.Contig1_All	2B	539	14.20	6.04	-1.23	0.01	0.0218398	*
		CL957.Contig2_All	2B	1783	0.38	0.15	-1.34	0.49	0.6552492	*
		CL957.Contig4_All	2B	632	0.47	10.95	4.54	0.00	9.09E-09	Up
		Unigene12145_All	3B	2464	25.36	32.31	0.35	0.00	0.0049963	*
		Unigene22590_All	1A	234	3.34	1.67	-1.00	0.66	0.7853515	*
		Unigene23522_All	1A	240	4.02	3.21	-0.32	0.89	0.9053714	*
		Unigene42115_All	1B	244	9.42	1.56	-2.59	0.05	0.1142363	*
		Unigene7554_All	5B	2524	0.11	0.10	-0.14	0.85	0.95598	*
C4H	K00487	CL1206.Contig3_All	3B	799	4.18	6.99	0.74	0.09	0.2015274	*
		CL1531.Contig2_All	3B	1764	0.16	0.01	-4.00	0.57	0.6925014	*
		CL2266.Contig1_All	7D	1709	15.44	1.84	-3.07	0.00	5.99E-21	Down
		CL2266.Contig2_All	7D	759	33.65	8.14	-2.05	0.00	1.42E-12	Down
		CL2266.Contig3_All	7D	1650	6.94	0.01	-9.44	0.00	1.50E-14	Down
		CL2266.Contig4_All	7D	1195	8.05	0.01	-9.65	0.00	7.02E-12	Down
		CL2950.Contig1_All	1A	1907	34.03	21.86	-0.64	0.00	3.89E-06	*
		CL2950.Contig2_All	1A	1863	37.18	23.78	-0.64	0.00	1.52E-06	*
		Unigene22796_All	7D	702	4.35	0.01	-8.76	0.00	0.0014982	*
		Unigene41891_All	2B	248	1.53	0.01	-7.26	0.57	0.7226877	*
C4L	K01904	CL2837.Contig2_All	6A	851	19.75	23.46	0.25	0.25	0.3955931	*
		CL2837.Contig3_All	6A	1938	17.00	28.15	0.73	0.00	1.47E-06	*

		Unigene17571_All	2B	333	0.01	3.01	8.23	0.03	0.0780737	*
		Unigene27494_All	6D	295	3.01	0.01	-8.23	0.16	0.308295	*
		Unigene34417_All	6AL	430	3.72	0.72	-2.37	0.09	0.2009526	*
		Unigene36842_All	6DL	263	1.41	0.01	-7.14	0.57	0.6939017	*
		Unigene45489_All	6AL	326	4.21	1.03	-2.03	0.18	0.3350635	*
		Unigene8785_All	4D	1047	12.30	10.12	-0.28	0.31	0.4728786	*
CHS	K00660	CL3476.Contig1_All	2A	1637	76.78	0.64	-6.91	0.00	5.06E-157	Down
		CL3476.Contig2_All	2A	350	8.17	0.01	-9.67	0.00	0.0046072	*
		Unigene20601_All	2A	231	0.01	8.50	9.73	0.00	0.0130452	*
		Unigene27805_All	6A	684	2.38	2.92	0.30	0.63	0.7521356	*
		Unigene28375_All	7B	310	2.25	0.01	-7.81	0.24	0.3994402	*
CHI	K01859	Unigene1730_All	5B	1027	39.47	3.44	-3.52	0.00	4.71E-35	Down
F3H	K00475	CL3859.Contig1_All	3B	1362	35.32	30.50	-0.21	0.12	0.2439016	*
		CL3859.Contig3_All	3B	1208	39.36	25.73	-0.61	0.00	0.0002202	*
F3'H	K05280	CL1550.Contig1_All	2B	2882	38.27	34.51	-0.15	0.09	0.1909169	*
		CL1550.Contig2_All	2B	2648	0.01	0.19	4.25	0.08	0.1812475	*
		CL1550.Contig3_All	2B	1137	64.19	29.88	-1.10	0.00	1.15E-15	Down
		CL1550.Contig4_All	2B	1828	46.90	34.40	-0.45	0.00	0.0001812	*
		CL2266.Contig3_All	7D	1650	6.94	0.01	-9.44	0.00	1.50E-14	Down
		CL451.Contig1_All	1A	216	5.65	0.01	-9.14	0.10	0.2149999	*
		CL451.Contig2_All	1A	204	2.06	0.01	-7.69	0.57	0.694883	*
		Unigene11783_All	4D	1607	14.48	15.58	0.11	0.57	0.6885706	*
		Unigene22796_All	7D	702	4.35	0.01	-8.76	0.00	0.0014982	*
		Unigene40620_All	6D	231	1.70	0.01	-7.41	0.57	0.7127757	*
		Unigene40621_All	7D	322	1.07	2.10	0.97	0.45	0.615652	*

		Unigene9374_All	1A	1634	15.74	0.64	-4.62	0.00	1.94E-27	Down
F3'5'H	K13083	CL1206.Contig2_All	3B	1695	19.03	10.85	-0.81	0.00	0.0001072	*
		CL5203.Contig1_All	1A	801	11.69	8.93	-0.39	0.22	0.3953497	*
		CL5203.Contig3_All	1A	380	0.64	8.76	3.77	0.00	0.0007297	Up
		Unigene40621_All	7D	322	1.07	2.10	0.97	0.45	0.615652	*
DFR	K13082	Unigene26027_All	3A	1292	62.35	1.03	-5.92	0.00	6.09E-95	Down
		Unigene8801_All	7D	1297	30.19	62.80	1.06	0.00	3.74E-17	Up
LDOX	K05277	CL6736.Contig1_All	1B	1298	0.11	0.01	-3.46	0.86	0.9123464	*
		Unigene27227_All	6A	553	6.61	2.67	-1.31	0.05	0.1330289	*
		Unigene46065_All	6A	318	1.63	0.01	-7.35	0.37	0.526484	*
UFGT	K12930	CL1063.Contig2_All	1B	422	8.75	11.10	0.34	0.48	0.6411141	*
		CL1845.Contig1_All	1B	447	3.19	4.82	0.60	0.39	0.5502	*
		Unigene4541_All	1A	675	7.24	11.42	0.66	0.08	0.1815293	*
MYB	K09422	CL1298.Contig1_All	7AS	1061	26.33	20.30	-0.38	0.04	0.1132643	*
		CL1298.Contig3_All	7AS	996	24.02	23.45	-0.03	0.81	0.92373	*
		CL4983.Contig1_All	4B	755	6.00	0.37	-4.02	0.00	0.0002111	Down
		CL4983.Contig2_All	4B	313	0.01	3.28	8.36	0.03	0.0776145	*
		CL5792.Contig1_All	6D	1123	31.07	21.47	-0.53	0.00	0.0085894	*
		CL5792.Contig2_All	6D	1145	69.60	52.66	-0.40	0.00	0.0012309	*
		Unigene15629_All	5D	1087	13.76	5.98	-1.20	0.00	0.0004375	Down
		Unigene19264_All	2B	324	0.01	6.25	9.29	0.00	0.0050478	*
		Unigene25731_All	5B	980	10.60	0.56	-4.24	0.00	2.65E-10	Down
		Unigene27555_All	1D	668	10.87	0.86	-3.66	0.00	2.04E-06	Down
		Unigene27556_All	4A	444	5.00	0.01	-8.97	0.00	0.0139174	*
		Unigene31225_All	4B	393	5.40	0.01	-9.08	0.01	0.0200507	*

		Unigene6024_All	4B	1085	22.37	3.99	-2.49	0.00	2.48E-15	Down
		Unigene6885_All	3B	830	0.17	0.01	-4.09	0.86	0.9312231	*
MYC	K13422	CL3336.Contig1_All	4D	1883	0.01	7.89	9.62	0.00	2.10E-25	Up
		CL3336.Contig2_All	4D	1918	0.01	19.14	10.90	0.00	5.49E-64	Up
		CL3336.Contig3_All	4D	1903	0.01	18.36	10.84	0.00	9.24E-61	Up
		CL3336.Contig4_All	4D	1868	0.29	7.56	4.70	0.00	1.24E-19	Up
		CL3336.Contig5_All	4D	1361	0.01	17.37	10.76	0.00	4.59E-40	Up
